# Pharmacokinetic profile and effect on bone markers and muscle strength of two daily dosage regimens of calcifediol in osteopenic/osteoporotic postmenopausal women

**DOI:** 10.1007/s40520-020-01779-7

**Published:** 2021-01-28

**Authors:** Stefano Gonnelli, Maria Dea Tomai Pitinca, Silvia Camarri, Barbara Lucani, Beatrice Franci, Ranuccio Nuti, Carla Caffarelli

**Affiliations:** Department of Medicine, Surgery and Neuroscience, Policlinico Le Scotte, University of Siena, Viale Bracci 2, 53100 Siena, Italy

**Keywords:** Calcifediol, Vitamin D deficiency, Osteoporosis, Muscle strength, Myostatin

## Abstract

**Background:**

At present, although cholecalciferol represents the form of vitamin D of choice for the treatment of vitamin D deficiency, there is a growing interest in calcifediol.

**Aims:**

This study aimed to evaluate the efficacy and the safety of two different daily doses of calcifediol.

**Methods:**

Fifty osteopenic/osteoporotic women with serum levels of 25-hydroxyvitamin D (25OHD) between 10 and 20 ng/ml were randomized to a 6-month treatment with oral calcifediol 20 µg/day (*n* = 25) or oral calcifediol 30 µg/day (*n* = 25). In all, we measured the time course of the levels of 25OHD and other biochemical parameters. Moreover, we evaluated handgrip strength and serum levels of myostatin.

**Results:**

The peak increase in 25OHD levels was reached after 90 days of treatment in group 1 (59.3 ng/ml) and after only 60 days in group 2 (72.3 ng/ml); thereafter in both groups, the levels of 25OHD showed a tendency towards stabilization. After 30 days, all the patients treated with 30 µg/day had values of 25OHD > 30 ng/ml. Handgrip strength showed a modest but progressive increase which reached the statistical significance in the 30 µg/day group. This latter group also presented a modest and non-significant decrease in serum levels of myostatin.

**Conclusions:**

Calcifediol is able to rapidly normalize the vitamin D deficiency, and the 30 µg daily dosage could be suggested in those patients who need to rapidly reach optimal 25OHD levels. Moreover, the 6-month treatment with calcifediol at a dose of 30 µg results in a modest but significant increase in upper limb strength.

## Introduction

At present, vitamin D insufficiency/deficiency represents an important health problem worldwide. An adequate vitamin D status, as assessed by 25-hydroxyvitamin D (25OHD) serum levels is of crucial importance for maintaining calcium/phosphate homeostasis and bone health.

The concentration of 25OHD, although subjected to seasonal variations, is currently used to determine vitamin D status and interpreted as a reflect of vitamin D storage of the body [1]. 25OHD is relatively stable in serum with a half-life of 2–3 weeks, while its activated form, 1,25-dihydroxyvitamin D (1,25(OH)2D), has a half-life of about 4–6 h only [2]. The definition of normality and deficiency for vitamin D serum levels is a heavily debated topic; while there is unanimous agreement that values of 25OHD of < 10 ng/ml (= 25 nmol/l) represent a condition of severe deficiency, a consensus for what could be considered "normal" value does not exist [1, 2].

In 2011, the Institute of Medicine defined deficiency, insufficiency and sufficiency values as < 12 ng/ml (30 nmol/l), between 12 and 20 ng/ml (30 and 50 nmol/l) and between 20 and 30 ng/ml (50 and 75 nmol/l), respectively [3]. Other Scientific Societies indicated that sufficiency levels could be based on values > 30 ng/ml and suggested as the optimal range values between 30 and 50 ng/ml (75 and 125 nmol/l) [4]. There is also a general agreement that in patients treated with drugs associated with a reduction in the risk of fragility fracture (anticatabolic and anabolic drug) the supplementation of vitamin D is of crucial importance [4]. Some studies reported that osteoporotic patients with a mean 25OHD ≥ 30 ng/ml had a substantially greater likelihood of maintaining bisphosphonate response [5, 6]; moreover, Peris et al. found that the probability of inadequate response to bisphosphonate was fourfold higher in postmenopausal women with 25OHD serum level < 30 ng/ml [7].

Cholecalciferol or vitamin D3 represents the most common form of supplementation used today. However, since the curve that describes the dose–response relationship is widely variable, being influenced by many factors, the main ones being represented by: extent of intestinal absorption, polymorphisms of the gene encoding the vitamin D-binding protein, gene polymorphisms which regulate the enzyme 25-hydroxylase and the amount of body fat, it is difficult to define the correct dose of vitamin D in individual patients [8]. Moreover, considering that long-term administration of 100 IU of cholecalciferol increases the value of 25OHD by about 1 ng/ml and the half-life of cholecalciferol, to reach a condition of vitamin sufficiency in a subject with basal values of 25OHD equal to 10 ng/ml, it is necessary to administer elevate dosages of vitamin D for several months [6, 9].

Since this time interval could be too prolonged in some cases (e.g. if drugs active on skeletal tissue are administered simultaneously) [6], a possible alternative could be to administer an initial loading dose (or bolus dose). However, recent studies have suggested that the administration bolus doses above 100,000 IU vitamin D should be avoided due to the possibility of increasing bone resorption markers and the risk of falls and fractures [4, 10].

Calcifediol administered orally could be an interesting alternative to cholecalciferol for the prevention or treatment of vitamin D deficiency. Calcifediol, due to a better absorption, a much shorter half-life and independence from hepatic hydroxylation, is able to increase circulating 25OHD levels more rapidly than cholecalciferol [8]. In particular, calcifediol is a more polar and soluble metabolite that display lower volume of distribution and less trapping by adipose tissue [11]. The pharmacokinetics and efficacy of calcifediol were evaluated in several randomized studies either double-blind [12–15] or open-label [16–19]. These studies were mainly carried out on the general population and on postmenopausal women, and only two [16, 17] were conducted on osteopenic/osteoporotic populations. Furthermore, the duration of most of these studies was too short to demonstrate the presence of any possible adverse events due to the use of calcifediol.

Moreover, one study also reported that postmenopausal women treated with calcifediol better maintained or improved their lower limb strength/function characteristics with respect to those treated with cholecalciferol [13].

The aim of this study was twofold: first, to evaluate the effect of a 6-month treatment with two different daily doses of calcifediol (20 µg and 30 µg) on serum levels of 25OHD, 1–25(OH)2D and bone markers in postmenopausal osteopenic/osteoporotic women; second, to assess the effect of these two dose regimens of calcifediol on muscle strength as evaluated by the handgrip test.

## Subjects and methods

### Population

A cohort of consecutive Caucasian postmenopausal osteopenic/osteoporotic women (age range 55–70 years) who applied to the Metabolic Bone Diseases Outpatient Clinic of the Department of Internal Medicine at the University Hospital of Siena (Italy), were considered for enrolment in this prospective, randomized, open-label, two-arm, parallel groups study. The participants had to be community-dwelling postmenopausal (years since menopause > 5) osteopenic/osteoporotic women, have 25-hydroxyvitamin D serum levels between 10 ng/mL (= 25 nmol/l) and 20 ng/ml (= 50 nmol/l), have a body mass index (BMI) between 18.6 and 29.9 kg/m^2^ and an adequate calcium intake. For all subjects, a detailed medical history was obtained and blood pressure, height and weight were measured in a standardized fashion. The daily dietary calcium intake was assessed by a validated Food-Frequency Questionnaire including foods that account for the majority of calcium in the Italian diet [20]. Moreover, all patients were instructed to maintain their usual diet during the whole study period. Exclusion criteria were history of major osteoporotic fractures (hip, vertebrae, wrist, humerus, pelvis), bone mineral density (BMD) at total hip or femoral neck < − 3.0 T-score, cancer, Paget’s disease of bone, malabsorption syndrome, sarcoidosis, hypercalciuria, hypercalcemia, parathyroid disorders, cardiac or liver failure, III–IV stage chronic kidney disease, alcohol abuse and treatments with drugs able to influence bone and mineral metabolism (estrogens, SERMS, glucocorticoids, levothyroxine, heparin, anticonvulsants, immunosuppressant and antiretroviral therapies and other drugs known to interfere with vitamin D metabolism). The patients previously treated with antiosteoporotic drugs (teriparatide, denosumab and bisphosphonates) and those who had been treated with cholecalciferol, calcifediol or active vitamin D analogues during the 6 months before the enrolment in the present study were also excluded.

Fifty patients met the eligibility criteria and were randomly assigned through a computer-based system to a 6-month treatment with oral calcifediol 20 µg (4 drops) daily (*n* = 25, group 1) or oral calcifediol 30 µg (6 drops) daily (*n* = 25, group 2). The randomization list was carried out by an independent pharmacist using a specific software. All patients were recommended to take the study drug in the morning (in fasting state). Participants agreed to refrain changing their dietary calcium intake and from using any vitamin D supplements during the 6-month study period.

For all patients, visits were scheduled at baseline and 15, 30, 60, 90 and 180 days after the start of the treatment to evaluate study parameters, compliance, adverse events and concomitant treatments. The study was conducted in the period September–May to avoid adequately the confounding influence of dermal synthesis of vitamin D.

An informed written consent was obtained from all participants, and the study was approved by the Institutional Review Board of Siena University Hospital and by the Italian drug agency (AIFA) (EUDRACT Code. 2015–005303-91).

### Laboratory evaluation

In all subjects, fasting venous blood samples were drawn at baseline and 15, 30, 60, 90 and 180 days afterwards to evaluate 25OHD, 1,25(OH)2D, parathyroid hormone (PTH), serum calcium (Ca), phosphate (P), creatinine (Cr), bone alkaline phosphatase (B-ALP), type I collagen β carboxy telopeptide (βCTX) and myostatin. At the same time points, 24-h urine samples were collected for the evaluation of calcium and creatinine. All samples were stored at − 80 °C while awaiting analysis, and then they were batched and measured in one assay.

Serum 25OHD was determined by a chemiluminescence immunoassay (LIAISON 25OHD Total Assay, DiaSorin Inc, Stillwater, MN, USA). In our institution, the intra- and inter-assay coefficients of variation were 6.8% and 9.2%, respectively. Serum1,25(OH)2D was assessed by chemiluminescence immunoassay (LIAISON XL 1,25-Dihydroxyvitamin D, DiaSorin Inc, Stillwater, MN, USA). In our institution, the intra- and inter-assay coefficients of variation were 4.1% and 5.3%, respectively. Serum B-ALP was measured by a chemiluminescence immunoassay method (LIAISON BAP Ostase, DiaSorin Inc., Stillwater, MN, USA). In our institution, the intra-and inter-assay coefficients of variation for B-ALP were 4.2% and 7.9%, respectively. Serum PTH was assessed by an immunoradiometric assay (Total Intact PTH Antibodies Lab. Inc.; Santee, CA, USA) and the intra- and inter-assay coefficients of variation were 3.6 and 4.9%, respectively. Serum βCTX was evaluated by an enzyme-linked immunoassay method (Immunodiagnostic Systems, Boldon, UK); in our institution the intra- and inter-assay coefficients of variation were 2.5% and 4.0%, respectively. Serum myostatin was determined by an ELISA method (Human Myostatin, Elisa Kit, My BioSource, San Diego, CA,). In our institution the intra- and inter-assay coefficients of variation were 5% and 8%, respectively. Serum and urinary calcium, phosphate and creatinine were determined by a colorimetric method (Cobas C311 analyzer, Roche Diagnostics, USA).

### Muscle strength evaluation at upper limb

At all visits, the maximal isometric contraction handgrip strength (HGS) in the dominant hand was measured using a digital hand dynamometer (DynEx; Akern/MD Systems, Florence, Italy). Using this device, sitting patients were tested with their shoulder adducted, the elbow flexed at 90° and forearm and wrist in neutral position; the mean value of three tests was taken into account. In women, HGS values < 16 kg indicate a condition of low muscle strength (dynapenia). At baseline body composition parameters (namely lean mass and fat mass) were measured in all subjects using a dual-energy X-ray absorptiometry device (Lunar Prodigy; GE Healthcare, Waukesah, WI) in conjunction with Encore 2002 software. All scans were performed by the same operator while the subjects were wearing light indoor clothing and no removable metal objects.

### Statistical analysis

All values were expressed as mean ± SD. The Kolmogorov–Smirnov test was used to verify the normality of the distribution of the outcome variables. Clinical data and initial values of the variables measured in the study groups were compared using Student’s *t* test and Mann–Whitney *U* test as appropriate. Categorical variables were compared by *χ*^2^ test or Fisher’s exact test, as appropriate. The associations between different parameters were tested by either Pearson’s correlation or Spearman’s correlation as appropriate.

ANOVA was used to test within and between intervention group effects of treatment, and the Bonferroni-adjusted *t* test was used for post-hoc analysis. All tests were two-sided, and *p* < 0.05 was considered statistically significant. All tests were performed using the SPSS statistical package for Windows version 16.0 (SPSS Inc., Chicago).

## Results

Forty-six patients (23 in the group 1 treated with calcifediol 20 µg and 23 in the group 2 treated with calcifediol 30 µg) completed the 6-month study period, and four patients withdrew from the study for problems unrelated to the study drugs (one for withdrawal of consent and three for unexpected logistical or health problems). Only the results from the women who completed the 6-month follow-up were analyzed and reported here. The baseline characteristics of the 46 women who completed the study period are shown in Table 1. There were no significant differences between the two groups for baseline demographic, clinical, biochemical, and densitometric characteristics. Also the mean values of upper limb muscle strength evaluated using a digital hand dynamometer were comparable.

**Table 1:** Demographic and clinical characteristics of the study population at baseline

	Calcifediol (20 µg/die) (*N* = 23)	Calcifediol (30 µg/die) (*N* = 23)
Age (years)	62.4 ± 7.4	61.5 ± 8.3
BMI (kg/m^2^)	26.1 ± 3.2	25.5 ± 4.0
Age of Menopause (years)	50.5 ± 3.3	49.7 ± 4.6
Calcium (mg/dl)	9.3 ± 0.3	9.4 ± 0.4
Phosphate (mg/dl)	3.5 ± 0.3	3.7 ± 0.4
Creatinine (mg/dl)	0.7 ± 0.1	0.7 ± 0.2
Urinary calcium (mg/24 h)	158.1 ± 70.4	149.5 ± 69.1
ALP (UI/L)	76.8 ± 13.4	76.9 ± 23.6
25OHD (ng/ml)	15.2 ± 4.7	16.2 ± 5.1
1,25(OH)D2 (pg/ml)	44.9 ± 11.8	45.5 ± 11.3
PTH (pg/ml)	42.6 ± 15.1	43.8 ± 14.4
B-ALP (µg/l)	15.1 ± 5.6	13.5 ± 4.3
β-CTX (ng/l)	459 ± 207	490 ± 120
LS-BMD (g/cm^2^)	0.967 ± 0.159	0.957 ± 0.107
LS T-score	− 1.47 ± 1.17	− 1.52 ± 0.79
TH-BMD (g/cm^2^)	0.835 ± 0.135	0.822 ± 0.084
TH T-score	− 1.18 ± 1.20	− 1.26 ± 0.75
FN-BMD (g/cm^2^)	0.744 ± 0.129	0.762 ± 0.112
FN T-score	− 1.76 ± 1.03	− 1.57 ± 0.66
Fat mass (kg)	28.9 ± 7.8	28.8 ± 6.9
Lean mass (kg)	37.9 ± 5.4	37.1 ± 5.2
Calcium Intake (mg/day)	684.0 ± 279.3	709.9 ± 322.1
Myostatin (ng/ml)	10.18 ± 5.91	11.07 ± 5.69
Right handgrip (kg)	18.47 ± 4.66	18.29 ± 4.86

**Fig. 1:**
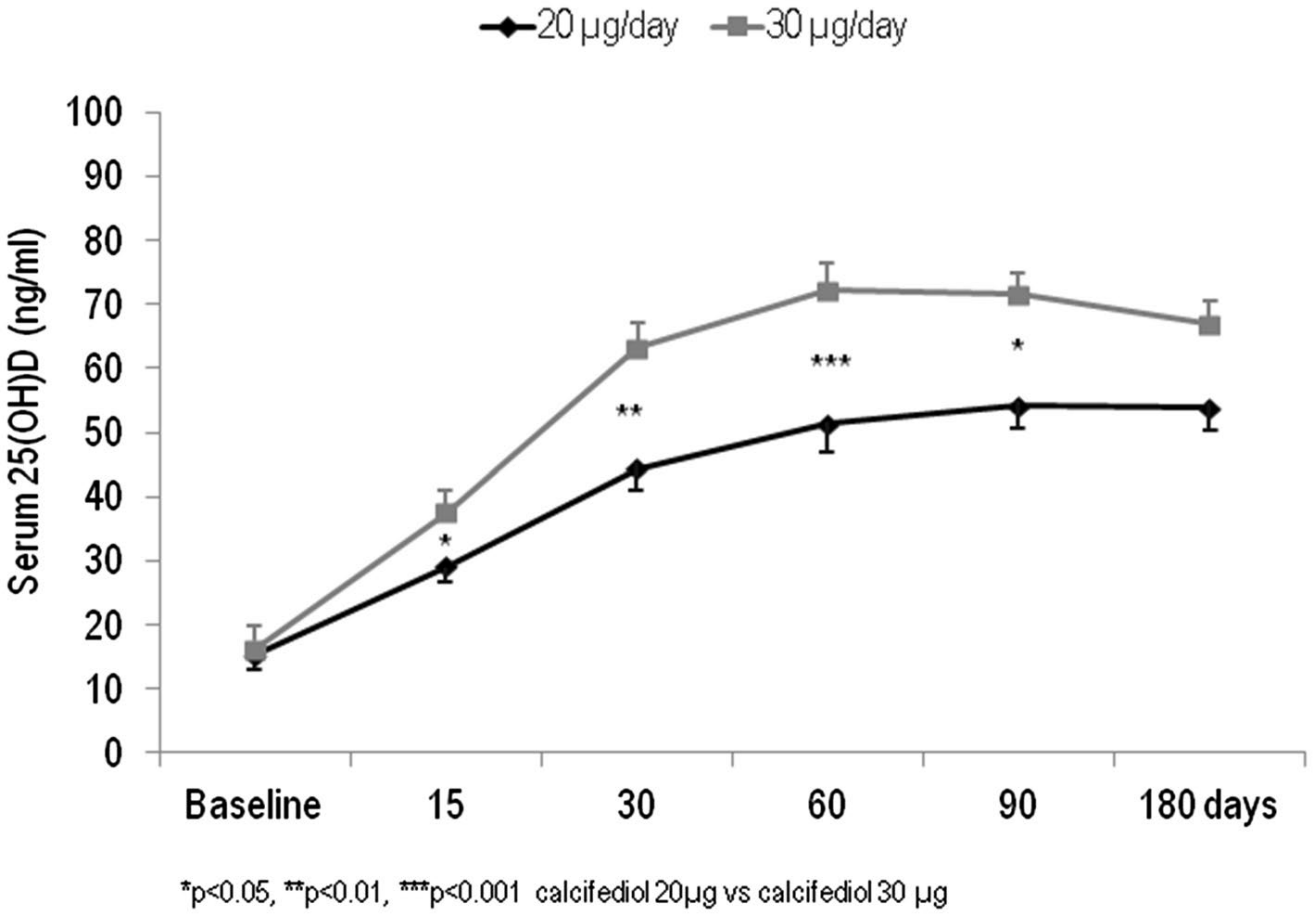
Mean values of 25(OH)D serum levels over time in participants grouped by different dose regiments of calcifediol

Figure 1 presents the changes in serum 25OHD levels per treatment group throughout the 6-month intervention period. At baseline the values of 25OHD were 15.2 ± 4.7 ng/ml in group 1 (20 µg/day calcifediol) and 16.2 ± 5.1 ng/ml in group 2 (30 µg/day calcifediol). After 15 and 30 days of treatment, the values of 25OHD increased to 28.1 ng/ml and 44.4 ng/ml in group 1 and to 40.5 ng/ml and 63.1 ng/ml in group 2. On day 15, all the women in group 2 and 19 women out of 23 in group 1 (82.6%) had crossed the 20 ng/ml threshold for 25OHD. After 30 days of treatment, 20 out of 23 (87.0%) of the patients treated with 20 µg/day and all those treated with 30 µg/day had values of 25OHD > 30 ng/ml. The peak increase in 25OHD levels was reached after 90 days of treatment in group 1 (59.3 ng/ml) and after only 60 days in group 2 patients (= 72.3 ng/ml). Furthermore, in both groups from the third to the sixth month, the levels of 25OHD showed a tendency towards stabilization or mild reduction (Fig. 1).

**Fig. 2:**
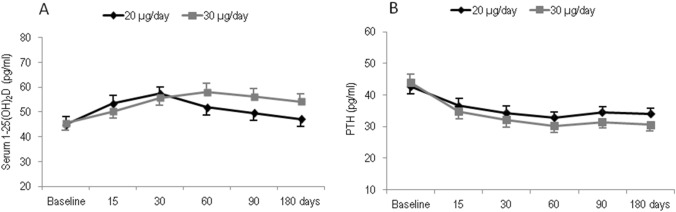
Mean values of 1,25(OH)D serum levels (**a**) and PTH serum levels (**b**) over time in participants grouped by different dose regiments of calcifediol

Figure 2a shows the time course of 1,25(OH)2D serum levels. From baseline to 30 days 1,25(OH)2D increased in both group 1 (+ 12.7 pg/ml) and group 2 (+ 11.2 pg/ml); afterwards, the levels of 1,25(OH)2D showed a tendency towards reduction in both groups. At the end of the study period, the levels of 1.25(OH)2D were higher in group 2 than in group 1 (54.2 pg/ml vs 47.1 pg/ml), but the difference was not statistically significant. Also PTH levels rapidly decreased in both groups (Fig. 2b). From baseline to 60 days the reduction in PTH levels was greater in group 2 than in group 1 (− 13.7 pg/ml vs − 9.8 pg/ml, respectively). At the end of the 6-month study period the values of PTH were 33.1 pg/ml in group 1 and 30.5 pg/ml in group 2 (Fig. 2b).

**Fig. 3:**
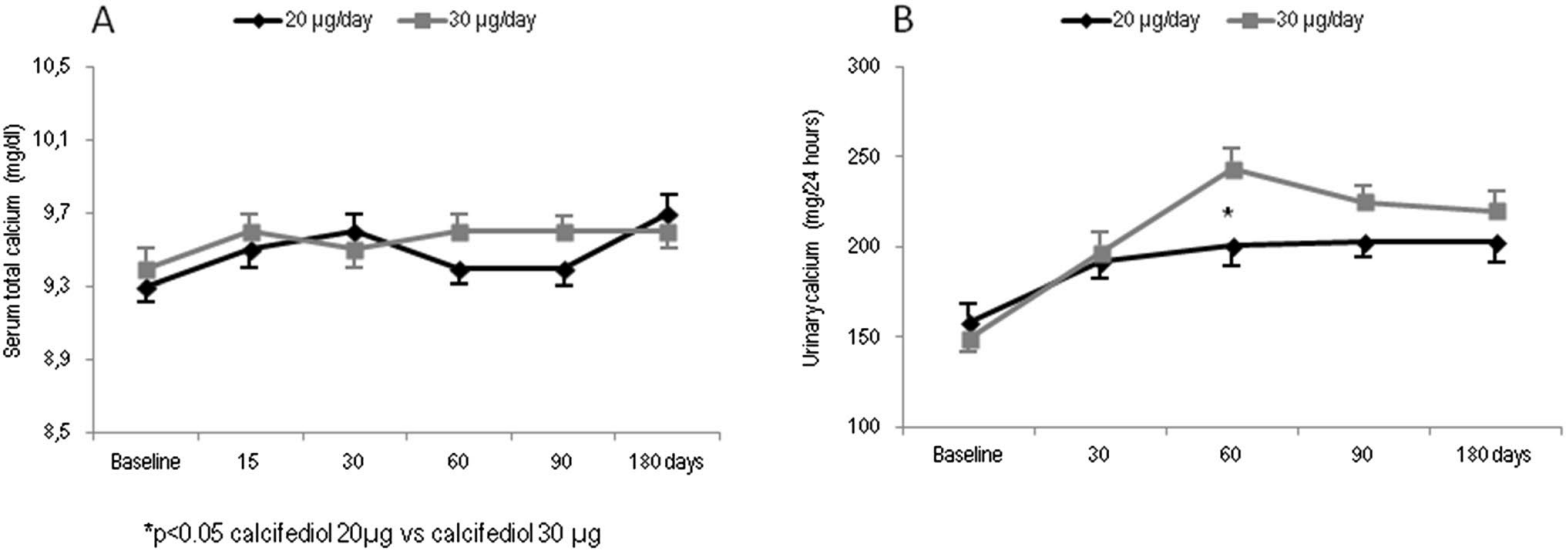
Mean values of calcium serum levels (**a**) and 24/h urinary calcium (**b**) over time in participants grouped by different dose regiments of calcifediol

Total serum calcium showed a tendency to increase in both groups, but no statistically significant differences between the two groups and versus baseline were observed (Fig. 3a). In both groups, the 24-h urinary calcium values were significantly higher with respect to baseline at all time points and peaked at month 2 (201.9 mg in group 1 and 241.9 mg in group 2, respectively) when the difference between the 2 groups resulted to be statistically significant (*p* < 0.05); subsequently, between 3 and 6 months of treatment, the 24-h urinary calcium values remained stable. Moreover, none of the patients in group 1 and only 1 in group 2 presented hypercalciuria borderline (i,e., urinary calcium 320 mg/24/h) (Fig. 3b). No significant differences with respect to baseline were observed during the study period for B-ALP and βCTX serum levels (data not shown).

**Fig. 4:**
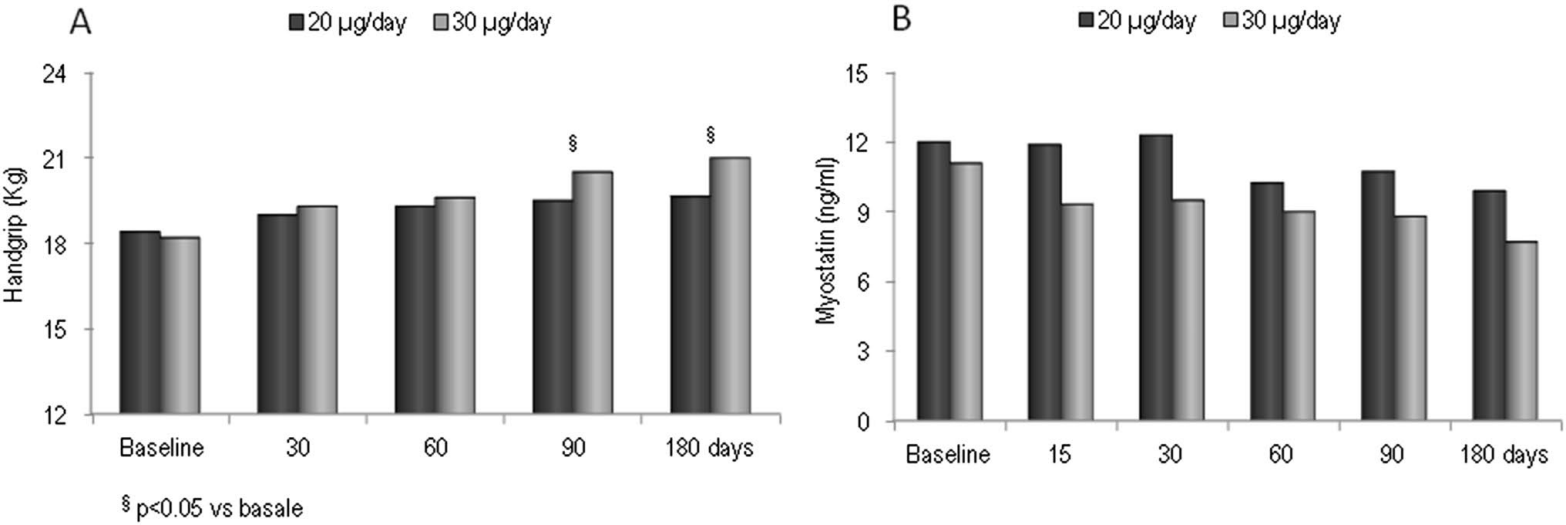
Mean values of handgrip (**a**) and myostatin serum levels (**b**) over time in participants grouped by different dose regiments of calcifediol

In both groups, the values of handgrip strength showed a modest but progressive increase (Fig. 4a). After 3 and 6 months, the values of HGS were significantly higher with respect to baseline (*p* < 0.05) in women treated with calcifediol 30 µg but not in those treated with calcifediol 20 µg (Fig. 4a). The serum values of myostatin remained substantially unchanged in the patients of group 1, whereas they showed a non-significant tendency towards reduction in those of group 2 (Fig. 4b).

## Discussion

Vitamin D deficiency represents a health problem of crucial importance worldwide due to its negative influence on musculoskeletal apparatus with a consequent increase in fragility fracture risk. At present, although the large majority of the Scientific Societies recommend cholecalciferol as the form of vitamin D of choice for the prevention and the treatment of vitamin D deficiency and suggest that calcifediol supplementation could be used only in few sub-populations of patients (such as those with hepatic insufficiency or intestinal malabsorption), there is a growing interest in calcifediol [4, 8, 11, 21].

To our knowledge, this is the first randomized study which evaluated the pharmakinetic profile of calcifediol given in a dosage of 30 µg to osteoporotic women. This study, in agreement with several previous reports [12–19], confirms the efficacy of two dose regimens of calcifediol (20 µg/day and 30 µg/day) to quickly correct vitamin D deficiency in postmenopausal osteopenic/osteoporotic women. After 30 days of treatment all the patients treated with 30 µg/day and 87% of those treated with 20 µg/day had serum values of 25OHD > 30 ng/ml. The rapidity in normalizing levels of serum 25OHD is of particular importance in those patients with high or imminent risk of fragility fractures who need to be treated with either denosumab or zoledronate or in those who are about to start glucocorticoids or aromatase inhibitors. Moreover, some studies have suggested the necessity for restoring 25OHD serum levels to threshold of 30 ng/ml prior to initiating antiresorptive drugs [5, 22]. Cholecalciferol and calcifediol, although being two chemically similar molecules and strictly related in terms of metabolism, present different pharmacokinetic and pharmacodynamic characteristics which may explain the differences in efficacy and safety. While calcifediol is rapidly absorbed and reaches the blood stream via the vena porta, the intestinal absorption of cholecalciferol is impaired in the case of intestinal fat malabsorption because of the need to be transported by chylomicrons to general circulation via the lymph pathway. Moreover, calcifediol presents lower affinity to fat tissue and lower elimination half-life with respect to cholecalciferol [8, 21]. Therefore, calcifediol, due to its rapidity in correcting vitamin D deficiency, could represent a possible alternative to the loading mega doses of cholecalciferol. At present, no consensus has been reached on the conversion factor to be used to calculate the equivalence between calcifediol and cholecalciferol; however, calcifediol is considered to be 2–4 times more potent than cholecalciferol; therefore, 1 µg of calcifediol could be equivalent to 80–160 IU of cholecalciferol [12, 17, 19].

In agreement with previous studies [18, 23], 1,25(OH)2D levels similarly increased in the first 30 days of treatment in both group 1 and group 2 (+ 12.7 pg/ml and + 11.2 pg/ml, respectively); afterwards, the levels of 1,25(OH)2D showed a tendency towards reduction. The observation of the tendency over time of serum levels of 25OHD and of 1,25(OH)2D to stabilize seems to suggest that the two dose regimens of calcifediol do not overwhelm the compensatory mechanisms of calcium homeostasis. Some studies have reported enhanced 24-hydroxylase expression (an enzyme responsible for calcitriol and 25OHD catabolism), in patients treated with calcifediol [1, 21]. Moreover, other studies reported that serum 24,25(OH)2D showed similar dose–response patterns to serum 25OHD, suggesting an activation of the catabolic pathway to regulate 1,25(OH)2D [15, 24].

Moreover, one key finding of this study was the confirmation about the long-term safety of calcifediol and the lack of toxic effects. According to previous studies [12, 13, 19] there was no case of hypercalcemia and only a transient case of borderline hypercalciuria.

Another interesting finding of this study was that supplementation with calcifediol seems able to improve the muscle strength of the upper limbs, as assessed by hand-grip test. However, the changes in hand-grip reached the statistical significance only in the group treated with the higher dosage; moreover, this latter group presented a modest and non-significant decrease in serum levels of myostatin, a member of the transforming growth factor-beta/bone morphogenetic protein super family, which may function as a potent inhibitor of skeletal muscle growth. The discovery that also human skeletal muscle cells express the vitamin D receptor (VDR) and that vitamin D metabolites directly interact with VDR promoting myogenic differentiation and decreasing the expression of myostatin has stimulated interest in the relationships between vitamin D and muscle [25, 26].

More recently, Girgis et al. reported that diet-induced vitamin D-deficient mice had significantly higher levels of myostatin and weaker grip strength than their controls [27].

Moreover, sarcopenic patients who sustained distal radius fractures presented in skeletal muscles lower gene expression of VDR and greater gene expression of myostatin compared to non-sarcopenic individuals [28]. However, the relationships between muscle strength, vitamin D and myostatin in humans have not yet been well defined [28].

Even though several studies have reported that vitamin D supplementation might increase muscle strength in subjects with severe vitamin D deficiency (25OHD < 10 ng/ml), the effectiveness of vitamin D supplementation on muscular performance is still being debated and there are conflicting results concerning the effect of vitamin D on muscle strength [29, 30]. Moreover, the meta-analysis by Beaudart et al. reported a small but significant positive effect of vitamin D supplementation on muscle strength without effect on muscle mass or muscle power [31]. Since calcifediol, unlike cholecalciferol, is able to bind to the muscle VDR we could speculate its greater effect on muscle. However, at present, only few studies have investigated the effects of calcifediol on muscle [12, 32, 33]. In particular, Bishoff-Ferrari et al. reported that in postmenopausal women a 4-month treatment with calcifediol (20 µg/day) induced a significantly greater (+ 17%) improvement in knee extension strength with respect to the treatment with cholecalciferol (800 IU/day) [13]. Instead, in the study by Vaes AMM carried out on older adults, a 6-month treatment with 10 µg/day of calcifediol did not change muscle strength and physical performance [15]. Another study carried out on hip fracture patients reported that vitamin D serum levels are associated with handgrip strength but not with muscle mass or length of hospital stay after hip fracture [34]. Another study carried out on elderly patients (70 years and older) with a prior fall event reported that a combined treatment with both calcifediol and cholecalciferol conferred no benefit on the prevention of functional decline and increased falls [32].

More recently, Iolascon et al. in a prospective study reported the effectiveness of calcifediol (20 µg/day for a 6-month period) in improving appendicular muscle strength and reducing the number of falls [35].

In conclusion, this study demonstrates that calcifediol, at daily doses of 20 and 30 µg, is able to rapidly normalize the vitamin D deficiency in osteopenic or osteoporotic postmenopausal women; in particular, the 30 µg daily dosage could be suggested in those patients who need to rapidly reach optimal 25OHD levels. This study also confirms the long-term safety of both 20 µg and 30 µg daily dose regimens of calcifediol. Moreover, this study shows that a 6-month treatment with calcifediol at a dose of 30 µg/day results in a modest but significant increase in upper limb strength. Further studies are warranted to assess muscle performance and strength as primary outcome of calcifediol supplementation and to address the possible role of myostatin.
